# Prosthetic Knee Joint Infection Caused by *Mycobacterium kansasii*

**DOI:** 10.5435/JAAOSGlobal-D-21-00183

**Published:** 2022-04-07

**Authors:** Suhas P. Dasari, Adam E. Hadro, Reena Singh, John C. Neilson

**Affiliations:** From the Department of Orthopaedic Surgery at the Medical College of Wisconsin, Wauwatosa, WI (Mr. Dasari, Dr. Hadro, and Dr. Neilson), and the Department of Pathology at the Medical College of Wisconsin, Wauwatosa, WI (Dr. Singh).

## Abstract

*Mycobacterium kansasii* is a nontuberculous mycobacterium that is a rare cause of prosthetic joint infections (PJIs). This case report presents a 58-year-old man who developed rapidly progressive arthritis after exposing his right knee to an unknown fluid at a microbial pharmaceutical company. Within a year, he underwent a right total knee arthroplasty (TKA). At 5 months postoperatively, he presented with pain and swelling of that knee. Imaging revealed extensive periprosthetic osteolysis with diffuse intracapsular and posterior extracapsular fluid collections. Multiple knee aspirates had negative cultures, and infectious laboratory test results were equivocal. Two years after his primary arthroplasty, the patient underwent posterior débridement and one-stage revision TKA with antibiotic cement. Synovial fluid mycobacterial cultures aspirated 2 weeks before the revision surgery became positive on postoperative day 1. PCR identified *M kansasii*. At 3 weeks postoperatively, intraoperative periprosthetic cultures grew mycobacterium. *M kansasii* was confirmed using mass spectrometry. Once susceptibilities returned, the patient was treated with targeted antimycobacterial therapy. This case report demonstrates the importance of considering atypical PJI in painful TKA with negative cultures and equivocal laboratory results. In the future, when there is concern for an atypical PJI, molecular diagnostic tools and mycobacterial cultures should be used before surgical intervention.

There has been a drastic increase in the number of joint arthroplasties done because improved techniques and implants have led to subsequent improvements in outcomes. Although most cases are successful, prosthetic joint infections (PJIs) complicate 1.7 to 2% of arthroplasty procedures.^1,2,3,4^ Culture-negative PJIs are relatively frequent and remain diagnostically challenging.^5^ Approximately 60% of PJIs are due to gram-positive cocci, but 5% to 35% of PJIs can remain culture-negative.^3,5^ Mycobacteria are a rare cause of PJI, making up zero to 0.6% of cases, with the majority caused by *Mycobacterium tuberculosis.*^6^ A mycobacterial PJI is difficult to manage because of its ability to adhere to implants, its ability form architecturally complex biofilms, and its intrinsic antibiotic resistance.^1,3^

Although *M tuberculosis* has been associated with up to 0.3% of PJI cases, nontuberculous mycobacterium (NTM) is an exceptionally rare cause of PJI.^5^ Wang et al^4^ identified only 16 cases of NTM total knee arthroplasty (TKA) PJIs. *Mycobacterium kansasii* is a slow growing NTM that is a relatively common cause of NTM pulmonary infection but is rarely involved in septic arthritis.^7^ Bernard et al^8^ reported a total of 50 cases of *M kansasii* septic arthritis in the literature and no cases of PJI. In 2006, Neuberger et al^7^ introduced the first case of a PJI due to *M kansasii.* This report presents the second case of an *M kansasii* PJI*.*

## Case

**Figure 1:**
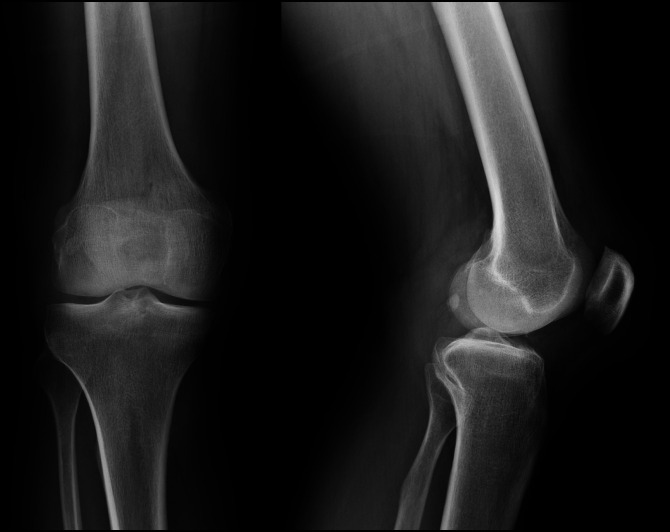
AP (left) and lateral (right) radiographs showing the patient's right knee before the development of his initial skin lesion over the right knee joint.

A 58-year-old man exposed his right knee to an unknown fluid at a microbial pharmaceutical company. He presented to the emergency department 1 month later with a painful, swollen right knee and an overlying pustule. His symptoms resolved with oral antibiotics. Baseline knee radiographs before this fluid exposure are shown in Figure 1.

**Figure 2:**
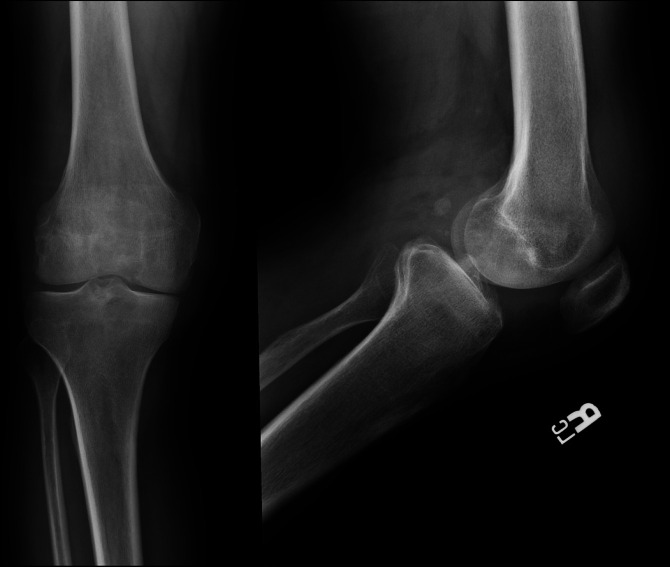
AP (left) and lateral (right) preoperative radiographs obtained 1 month before the index total knee arthroplasty procedure and roughly 1 year after the patient's initial development of the skin lesion demonstrating rapidly progressive arthritis.

**Figure 3:**
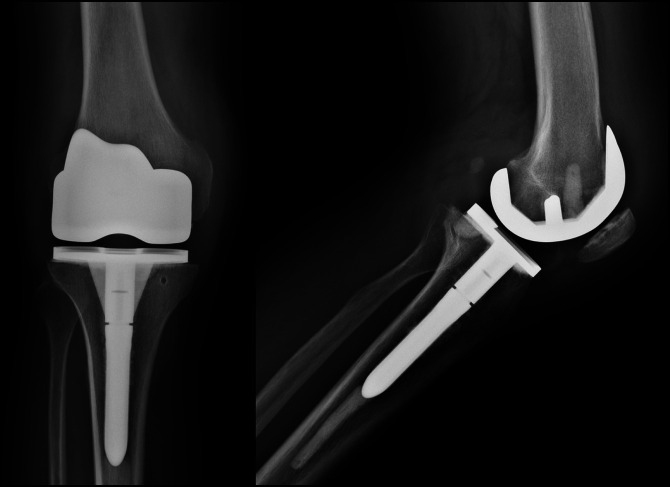
AP (left) and lateral (right) radiographs showing the right knee 3 months after index total knee arthroplasty.

Several months later, the pain and swelling recurred. An MRI demonstrated bursitis. He underwent a bursectomy that relieved his symptoms until 2 months later, when he once again developed pain and swelling. Repeat radiographs obtained 1 year after his initial presentation demonstrated evidence of rapidly progressive arthritis (Figure 2). He underwent a right TKA at an outside hospital (Figure 3). Per outside records, there was notable joint destruction with hypertrophied synovium and poor-quality bone. Synovial biopsy showed noncaseating granulomas with negative acid-fast and fungal stains. The patient followed an uncomplicated postoperative course until 5 months postoperatively, when he began to experience intermittent right knee swelling. Infectious workup was negative, so no further intervention was pursued.

Two years later, the patient presented with worsening pain, swelling, and limited range of motion of his right knee. Radiographs revealed periprosthetic osteolysis. An ultrasonography-guided aspiration was bloody with 1033 WBCs, 70% neutrophils, 29% lymphocytes, and negative synovial fluid cultures. An MRI demonstrated complex fluid collections around the knee and proximal calf. Pigmented villonodular synovitis (PVNS) was suspected, and the patient was referred for additional treatment.

**Figure 4:**
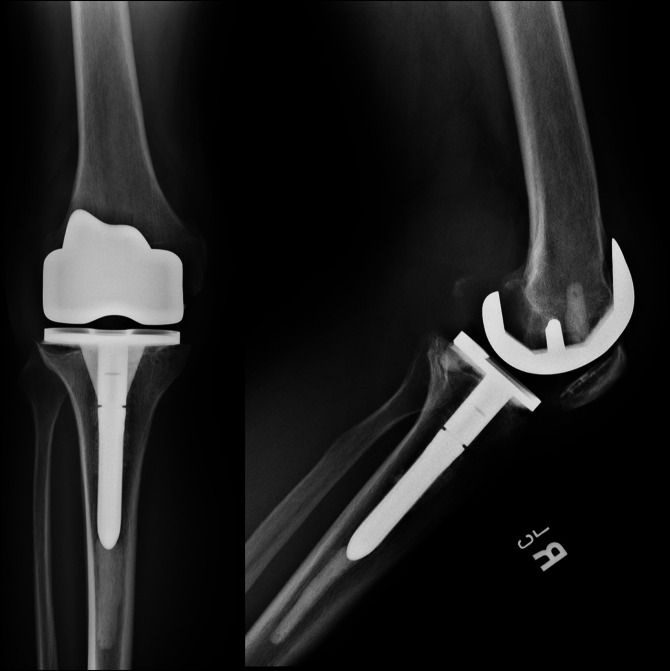
AP (left) and lateral (right) radiographs showing the right knee 2 years after the initial total knee arthroplasty and 2 months before one-stage revision. Extensive osteolysis is seen around the femoral and tibial implants.

**Figure 5:**
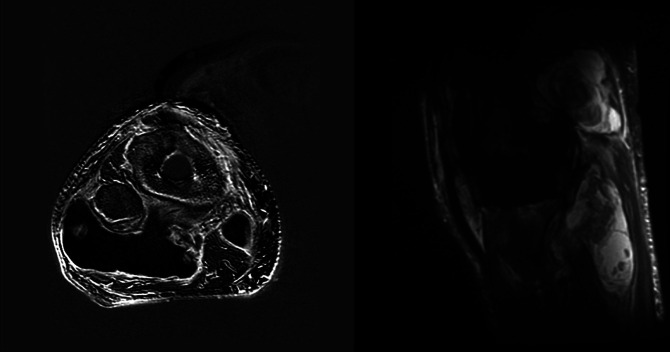
Radiograph showing preoperative metal suppression MRI (axial left and sagittal right) demonstrating abnormal signal around the total knee arthroplasty implants with associated large fluid collections containing heterogenous debris.

**Figure 6:**
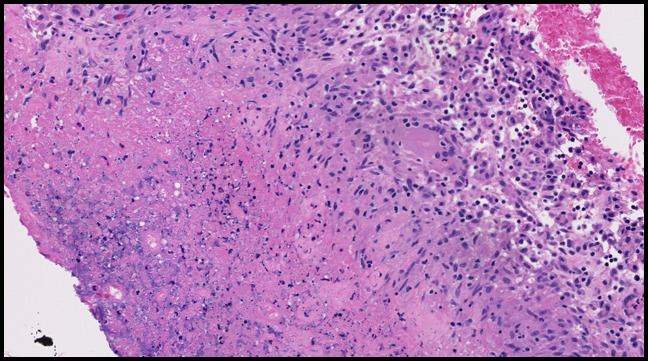
Photograph showing histologic examination of the preoperative ultrasonography-guided synovial biopsy demonstrated necrotic tissue that was bordered by lymphohistiocytic inflammation. This was not diagnostic for pigmented villonodular synovitis.

Repeat radiographs revealed progressive osteolysis (Figure 4). Infectious laboratory results were repeated and are presented in Table 1. The patient's c-reactive protein (CRP) was mildly elevated but stable compared with that a year earlier. The patient's presentation was concerning for PVNS, and a metal suppression MRI was ordered. The MRI demonstrated extensive extracapsular and intracapsular fluid collections and diffuse osteolysis (Figure 5). Because the MRI was not diagnostic of PVNS, a repeat ultrasonography-guided aspiration and biopsy were done. The fluid and tissue cultures were initially negative, but the WBC count was 13,260 with 93% neutrophils and 1% lymphocytes. Biopsy was not diagnostic of PVNS (Figure 6). A Kinyoun stain was negative for acid-fast bacilli (AFB).

**Table 1:** Pertinent Laboratory Values

Laboratory	Value
CBC	
Leukocyte count	8.5 K/μL
Hemoglobin	14.6 g/dL
Platelet count	294 K/μL
Inflammatory markers	
CRP	21.87 mg/L
ESR	15 mm/hr
Knee fluid aspiration analysis (2 months preoperatively)	
Gross findings	Red fluid and no synovial crystals
Cultures	Negative and sterile
Total nucleated cell count	1033 cells/microliters
Neutrophil count	70%
Lymphocyte count	29%
Knee fluid aspiration analysis (2 weeks preoperatively)	
Gross findings	Red fluid and no synovial crystals
Cultures	Initially negative but grew mycobacterium (2 weeks later)
AFB smears	Negative Kinyoun, Fite, and modified Auramine-O stains
PCR	*M. kansasii* (4 weeks later)
WBC	1,3260 cells/μL
Neutrophils	93%
Lymphocytes	1%
Monocytes	6%

AFB = acid-fast bacilli, CRP = C-reactive protein, ESR = Erythrocye Sedimentation Rate, PCR = Polymerase Chain Reaction, WBC = white blood cell count

**Figure 7:**
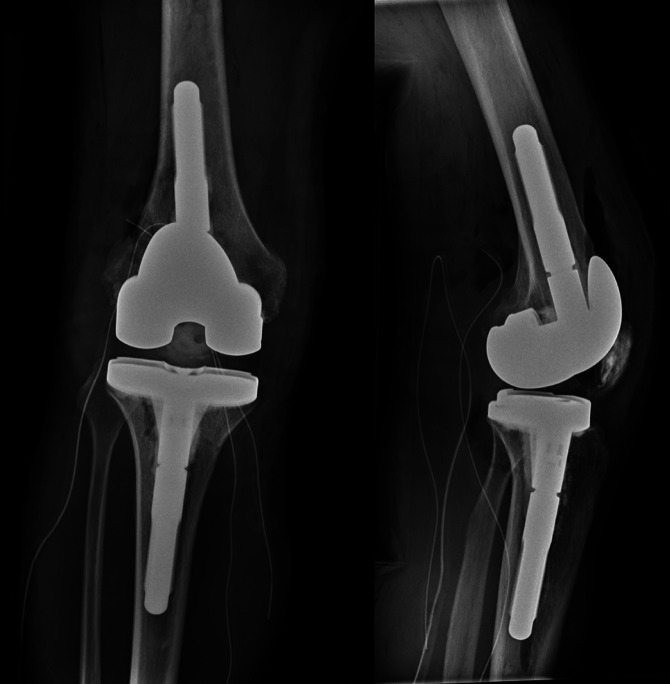
AP (left) and lateral (right) postoperative radiographs showing right knee revision total knee arthroplasty.

The patient elected to proceed with débridement and one-stage revision of his right TKA with antibiotic cement. This began with a posterior approach to the knee, where multiple extracapsular compartments of necrotic-appearing tissue were débrided. The patient was turned supine, and medial parapatellar arthrotomy was made. A large amount of intra-articular necrotic tissue and osteolysis were noted. A thorough synovectomy and débridement were done. The femoral, tibial, and patellar implants were grossly loose with extensive bone loss. After removal of the implants, a revision TKA with antibiotic cement was done. Postoperative radiographs are shown in Figure 7. Multiple periprosthetic samples were obtained for culture and histology.

**Figure 8:**
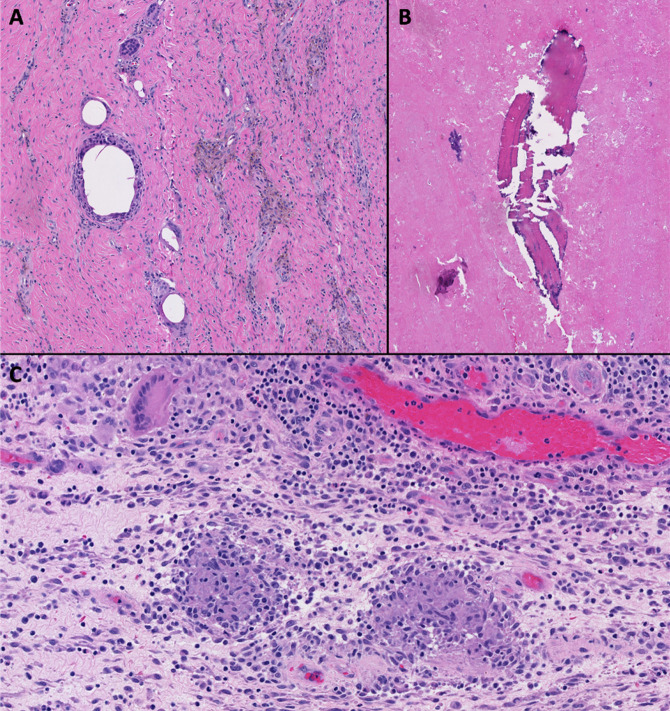
Photographs showing histologic examination of the intraoperative periprosthetic tissue samples demonstrating detritic synovitis (**A**), abundant necrotic tissue with osteolysis (**B**), and noncaseating granulomatous inflammation (**C**). The samples were negative for Kinyoun and Fite stains. There were no features suggestive of pigmented villonodular synovitis.

On postoperative day 1, the patient's mycobacterial cultures from the preoperative ultrasonography-guided biopsy grew AFB. Histological examination of the intraoperative periprosthetic tissue samples showed osteolysis, detritic synovitis, and noncaseating granulomatous inflammation (Figure 8). PCR identified *M kansasii* 2 weeks later. His chest imaging demonstrated no lung lesions. At 3 weeks postoperatively, intraoperative cultures grew mycobacterium despite modified Auramine-O stains remaining negative. *Mycobacterium kansasii* was confirmed using mass spectrometry. The patient was started on daily azithromycin 500 mg, ethambutol 1,200 mg, and rifampin 600 mg. At 7 weeks, the isolate was found to be rifampin-sensitive and ethambutol-sensitive, so his treatment continued unchanged.

At his most recent follow-up, he was 8 months postoperatively. His ESR had normalized, and CRP was down trending. There was no concern for infection based on clinical examination, and the patient demonstrated painless ambulation, minimal swelling, and no erythema. As a result, antimicrobial therapy was discontinued. Currently, the patient is working full time without restrictions and has no functional limitations.

## Discussion

This is the second reported case of *M kansasii* PJI in the literature. The patient initially presented with multiple negative cultures and equivocal laboratory results, raising suspicion for a synovial process. The final diagnosis of *M kansasii* demonstrates the importance of obtaining mycobacterial cultures and molecular tests in abnormal cases of painful TKAs.

Neuberger et al^7^ presented the only other case of *M kansasii* PJI. Their patient was an 82-year-old man who presented 6 years after his TKA with 6 months of pain. The patient was afebrile with equivocal laboratory results. A diagnostic knee arthroscopy noted loosening of the implants and periprosthetic tissue necrosis. All synovial fluid cultures were negative. Synovial histology demonstrated granuloma formation and acid-fast staining. They began empiric antimycobacterial treatment, but when PCR identified *M kansasii*, treatment was changed to ethambutol, rifampin, clarithromycin, and ofloxacin. The patient failed medical therapy and ultimately underwent arthrodesis. Most late-occurring PJIs are a result of hematogenous seeding or, in the case of *M tuberculosis* PJI, reactivation. By contrast, *M kansasii* normally causes chronic indolent infections. As such, Neuberger et al^7^ concluded that the most likely etiology of this patient's infection was from direct inoculation from unnoticed skin trauma.

Unlike the case discussed earlier, the patient in this report likely had an infection of his native joint before his TKA. The etiology of transmission was likely through the pustule that formed over his knee. Percutaneous transmission into the joint is the most likely pathway for this patient with normal chest imaging because the second most frequent organ involved in an *M kansasii* infection is the skin.^9^ This infection would explain the rapid degenerative joint disease resulting in primary TKA. Both cases had several sterile cultures and equivocal laboratory results that gave little initial suspicion for an infectious process. This contributed to the morbidity in each case because intraoperative findings showed extensive joint destruction before proper intervention was initiated. From these cases, it is evident that the difficulty in diagnosing *M kansasii* PJI can lead to delayed care. Thus, atypical pathogens need to be considered in culture-negative, painful TKAs with notable joint destruction.

Synovial tissue and fluid cultures are routinely obtained when PJI is suspected. Unfortunately, routine bacterial cultures are not ideal for mycobacterial isolation and can lead to false negatives and delayed treatment.^3^ In these cases with negative cultures, a neutrophil abundance tends to be the only nonspecific diagnostic finding for mycobacterial PJI.^7^ As such, when PJI is suspected and routine cultures are negative, specific mycobacterial cultures should be considered.^4^

Previous case reports have shown that periprosthetic tissue cultures are highly sensitive in the diagnosis of mycobacterial PJI.^3^ If PJI is suspected but routine cultures are negative, periprosthetic tissue samples should be acquired for mycobacterial culture.^3^ Unfortunately, even with proper tissue cultures, false negatives and delayed results are common. If clinical suspicion remains high for infection, molecular methods of identification should be explored.^7^ Despite the efficacy of molecular identification, tissue cultures are still required for antibiotic sensitivities. Thus, combining molecular identification, periprosthetic tissue cultures, and standard synovial cultures may be the best combination of diagnostic tests when there is concern for an atypical PJI.

The benchmark surgical treatment of fungal and late bacterial PJIs is two-stage revision.^3,7^ Although two-stage revisions have proven to be widely successful in treating rapid growing mycobacterial PJIs, the optimal surgical treatment of slow growing mycobacterial PJIs, such as *M kansasii*, is unclear.^3^ Neuberger et al^7^ opted to do an arthrodesis because of the unclear treatment protocol. Regarding other slow growing NTM PJIs, two-stage revision has been successful. There are examples in total hip arthroplasty literature of two-stage revisions being done successfully for *Mycobacterium avium* PJIs.^10,11^ Falola et al^12^ presented an interesting case of *M kansasii* native joint septic arthritis. The patient was treated with serial arthroscopic débridements and antibiotic therapy. Three years after her initial débridement, the patient successfully underwent TKA. This case report provides evidence that a two-stage revision for *M kansasii* PJI could be successful if the infection is treated appropriately.

In this report, *M kansasii* was not identified until after a one-stage revision TKA was done. If there is suspicion for an atypical, culture-negative PJI, then mycobacterial cultures should be done because routine bacterial cultures and AFB smears can be unreliable in identifying *M kansasii.* As molecular methods for identification become more routine in clinical practice, they should also be considered in painful TKAs with negative cultures and an unclear diagnosis. In the future, molecular diagnostic tools and mycobacterial cultures should be used before surgical intervention when there is concern for an atypical PJI.
